# Albumin-based drug delivery: harnessing nature to cure disease

**DOI:** 10.1186/s40591-016-0048-8

**Published:** 2016-02-27

**Authors:** Maja Thim Larsen, Matthias Kuhlmann, Michael Lykke Hvam, Kenneth A. Howard

**Affiliations:** Interdisciplinary Nanoscience Center (iNANO), Department of Molecular Biology and Genetics, University of Aarhus, Aarhus, Denmark

**Keywords:** Human serum albumin (HSA), Drugs, Albumin-binding, Albumin fusions, Half-life extension, Intracellular delivery, Neonatal Fc receptor (FcRn), Molecular medicine, Targeted drug delivery

## Abstract

The effectiveness of a drug is dependent on accumulation at the site of action at therapeutic levels, however, challenges such as rapid renal clearance, degradation or non-specific accumulation requires drug delivery enabling technologies. Albumin is a natural transport protein with multiple ligand binding sites, cellular receptor engagement, and a long circulatory half-life due to interaction with the recycling neonatal Fc receptor. Exploitation of these properties promotes albumin as an attractive candidate for half-life extension and targeted intracellular delivery of drugs attached by covalent conjugation, genetic fusions, association or ligand-mediated association. This review will give an overview of albumin-based products with focus on the natural biological properties and molecular interactions that can be harnessed for the design of a next-generation drug delivery platform.

## Background

The therapeutic efficiency of a drug is dependent on the availability at the target site at a concentration and frequency that maximises the therapeutic action and minimizes side-effects to the patient. Therapeutic drugs are often low-molecular weight molecules that result in non-specific distribution, with a molecular weight below the renal filtration threshold resulting in rapid renal clearance and concomitant short plasma circulatory time [1, 2].

Drug delivery technology has been utilised to overcome these obstacles. The standard method to extend the circulatory half-life of drugs, particularly peptide and protein-based, is by PEGylation using poly (ethylene glycol) (PEG) conjugation [3]. The PEGylation approach for drug delivery applications has proved to be effective with a large number of marketed drugs, for example, Adagen® (pegademase bovine) and Pegasys® (PEG-interferon alfa-2α) [4]. Drawbacks to PEGylation, however, include accumulation of high molecular weight PEG in tissues such as the liver [5] and the necessity for chemical conjugation of the drug. An alternative strategy is incorporation in nanoscale carriers (nanocarriers) of a size range that enables transit across tissue and cellular barriers [6]. Examples include liposomes, polymeric nanoparticles, dendrimers, and solid lipid nanoparticles [6–9]. A requirement for complex designs that includes surface engineering to reduce host foreign body responses, whilst maintaining cellular targeting capabilities, and possible toxicological issues due to non-specific accumulation of synthetic material would seemingly restrict clinical application in the short-term. This is exemplified by the limited number of nanocarrier-based marketed products. Albumin is an attractive next-generation “self” drug delivery approach. It is the most abundant plasma protein involved in transport of nutrients in the body facilitated by its multiple binding sites and circulatory half-life of ~19 days [10]. It is crucial, however, to understand its biological interactions in order to harness its properties towards drug delivery solutions.

### Biological properties of albumin

Albumin is the most abundant plasma protein in human blood (35–50 g/L human serum) with a molecular weight of 66.5 kDa [11]. It is synthesised in the liver hepatocytes with ~ 10–15 g of albumin produced and released into the vascular space daily [10, 12]. Circulation in the blood proceeds for an extended period of ~ 19 days [10, 13, 14]. This long half-life is thought mainly due to neonatal Fc receptor (FcRn)-mediated recycling, and the Megalin/Cubilin-complex rescue from renal clearance. Termination of the circulation is typically caused by catabolism of albumin in organs such as the skin and muscles [2, 12]. Modifications of albumin, for instance by non-enzymatic glycosylation, is thought to trigger lysosomal degradation [10, 15, 16]. Albumin contains multiple hydrophobic binding pockets and naturally serves as a transporter of a variety of different ligands such as fatty acids and steroids as well as different drugs [10]. Furthermore, the surface of albumin is negatively charged [10] making it highly water-soluble.

### Structure, domains and binding sites

**Fig. 1:**
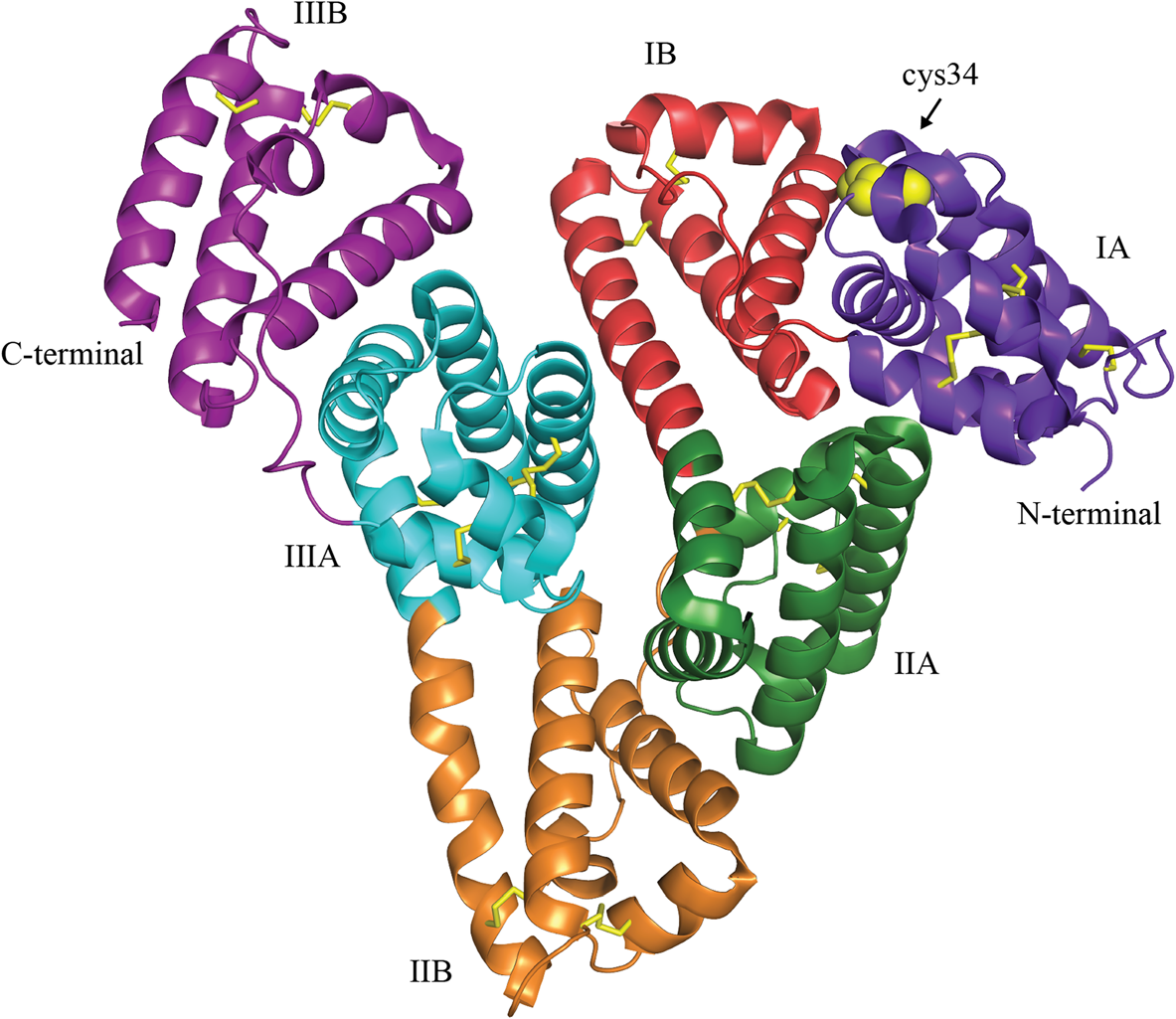
Crystal structure of human serum albumin. The illustration shows the tertiary structure of human serum albumin in complex with stearic acid (PDB 1e7e). The three domains of albumin are shown in purple (IA), red (IB), green (IIA), orange (IIB), blue (IIIA), and violet (IIIB). Yellow sticks depicture disulfide bridges, and yellow spheres highlight the available cysteine 34 in domain IA

The overall three-dimensional structure of human serum albumin (HSA), shown by X-ray crystallography, is heart-shaped (Fig. 1) [17]. Structurally, albumin consists of three homologous domains I, II, and III. Each domain contains two sub-domains (A and B), which contains 4 and 6 α-helices, respectively. The two main drug binding sites are named Sudlow site I and Sudlow site II [18]. Site I, positioned in subdomain IIA, reversibly binds the anticoagulant drug warfarin [19, 20]. In the subdomain IIIA Sudlow Site II is located. It is known as the benzodiazepine binding site and diazepam, which is used in the treatment of anxiety, binds with high affinity [19]. Site I and site II are the primary binding sites though it has been found that some drugs bind elsewhere in the protein [18, 21, 22].

Drugs and drug metabolites can also bind covalently to albumin. Glucuronidation of drugs as part of metabolism, often occurs to drugs having a carboxylic acid group resulting in acid glucuronides [19]. These acid glucuronide metabolites can bind covalently to HSA [23]. This can occur by nucleophilic attack from NH_2_, OH or SH in a protein to the acyl carbon of the glucuronide, giving a covalent attachment of drug to protein without retention of the glucuronide moiety. Another mechanism is the migration of the acyl group from position 1 in the sugar ring to 2, 3, or 4 position leading to tautomerism of the sugar ring. Aldehyde in the open tautomer structure reacts with a lysine group in the protein resulting in a covalent attachment of drug to protein with a glucuronic acid in between [19, 23, 24]. Covalent binding to albumin will naturally affect the clearance and metabolic destiny of such drugs. Drug metabolites such as furosemide, salicylic acid, and Nonsteroidal Anti-Inflammatory Drugs (NSAIDs) like ibuprofen react covalently with HSA [19].

Albumin contains 35 cysteine residues of which 34 form disulfide bridges internally in the structure. These contribute to the high stability of albumin. The availability of a free cysteine residue at position 34 (cys34) for covalent attachment of drugs is an attractive feature for drug delivery as it holds a free thiol group (−SH) accounting for 80 % of thiols in the plasma [17]. Cys34 is located on the outer surface of albumin distant from the main interior drug binding sites and has, therefore, been a focus for covalent conjugation of drugs [11, 25, 26].

### Albumin cellular receptors and engagement

Interaction with cellular receptors is responsible for albumin’s recycling, cellular transcytosis and hyphenate if word on two lines. Receptors include glycoproteins Gp60, Gp30 and Gp18, a secreted protein acidic and rich in cysteine (SPARC), the Megalin/Cubilin complex, and the neonatal Fc receptor (FcRn) [27–33]. Understanding the interaction with these cellular receptors is crucial for specific delivery of drug cargoes.

### Gp60 receptor

The Gp60 receptor, named because its molecular size of 60 kDa, also referred to as albondin, is a vascular endothelial membrane protein, which acts to increase membrane permeability for receptor-mediated uptake of circulating proteins [32, 34–38]. Binding of proteins such as albumin to the Gp60 receptor is proposed to activate the membrane protein caveolin-1, which induces the formation of a caveolae vesicle. The caveolae then migrate through the cytoplasm, fuses with the basolateral membrane and releases material from the caveolae into the interstitium. Gp60, therefore, is thought to facilitate cellular transcytosis of albumin and redirect albumin from lysosomal degradation [36–43].

In 1986 work from Ghitescu et al. confirmed albumin-binding surface receptor engagement in capillary endothelium in mouse lung, heart and diaphragm by showing albumin-gold complexes were adsorbed at specific bindings sites associated with the plasmalemmal vesicles [44]. Work by Schnitzer and Oh, showed ~ 50 % of albumin transport was facilitated by binding to Gp60, while fluid-phase transport via vesicles or transport through intercellular junctions, performed the remaining transport [36, 40, 45]. This was found by in situ and in vitro studies of albumin transport across lung microvascular endothelium. Albumin binding to the cell surface was almost completely inhibited by anti-Gp60 antibodies [40, 46].

### Secreted protein, acidic and rich in cysteine (SPARC) receptor

Secreted protein, acidic and rich in cysteine (SPARC) also known as osteonectin or basement-membrane 40, is an albumin binding protein located in the extracellular matrix and is expressed by a variety of cells including fibroblasts and endothelial cells and associated with tissue growth and cell movement and/or proliferation [47–53]. SPARC has been hypothesized to enhance tumour uptake of an albumin-based nanoparticle system of nab-paclitaxel (Abraxane®) though direct evidence remains to be elucidated [54].

### Gp18 and Gp30 receptor

The Gp18 and Gp30 are cell surface glycoproteins with molecular weights of 18 and 30 kDa, respectively. Gp18 and Gp30 are expressed in endothelium cell membranes, in particular in the liver [55] and peritoneal macrophages [10, 56]. Whilst Gp60 serves to rescue albumin from degradation, it has been shown that Gp18 and Gp30 bind to modified albumin, for instance gold-labelled albumin or formaldehyde-treated albumin [27, 31, 34, 36, 42, 45]. Gp18 and Gp30 will then direct the modified albumin to lysosomal degradation, possibly as a safety mechanism to remove old, damaged or altered albumin [40, 42, 45]. This was demonstrated by the study of Schnitzer et al. using a cell-based study of rat epididymal fat pads by investigating binding, uptake and degradation [42]. Albumin modified by formaldehyde, maleic anhydride or gold-attachment was shown to bind Gp18 and Gp30 with higher affinity than native albumin [42]. Modification of native albumin is thought to occur through oxidation or non-enzymatic glycosylation as a means of protection or simply due to normal aging or a disease-mediated reaction, for instance such as oxidation from inflammation or hyperglycation in diabetes [10, 40, 45, 57]. Hence, it appears that Gp18 and Gp30 are important for degradation of modified albumin, as altered albumin not only binds to Gp18 and Gp30 but are also internalized and degraded [42, 46]. Native albumin does not avidly bind to the Gp18 and Gp30 receptors, but binds to the aforementioned Gp60 receptor, which is responsible for transcytosis of albumin through endothelium [10]. Investigations of albumin interactions with Gp18 and Gp30 receptors have not been extensively explored, yet it has been shown that modified albumin is degraded faster than native albumin [10, 45] and that chemically modified bovine serum albumin (BSA) shows 1000-fold higher affinity for Gp18 and Gp30 compared to native bovine serum albumin [31]. In summary, these results suggest that the receptors Gp18 and Gp30 are responsible for the degradation of modified albumins and are, therefore, known as scavenger receptors.

### Megalin/Cubilin receptor

Cubilin is a glycoprotein expressed in the apical endocytic compartments of kidney proximal tubules, anchored to the membrane at the N-terminal by a α-helix. Cubilin lacks a transmembrane segment as well as a cytoplasmic domain, therefore, it depends on another membrane protein, Megalin, to facilitate endocytosis. Megalin has an extracellular domain, a transmembrane segment as well as a cytoplasmic tail. The binding site for albumin on Megalin, to our knowledge, has not been identified, yet, the functional role of the Megalin/Cubilin complex in reabsorption in the kidneys has been extensively studied. Reabsorption of filtered proteins in the kidney occurs by receptor-mediated endocytosis in the hyphenate if the word on two lines tubule. The receptors responsible for mediating the reabsorption are Cubilin and Megalin, both shown to bind albumin [29, 30]. As albumin binds to Cubilin and Megalin, it is likely that the Megalin/Cubilin complex is responsible for the receptor-mediated endocytosis and rescuing of albumin from renal excretion. Studies in Cubilin-deficient mice, as well as in humans with a mutation in a Cubilin gene [58], show a decrease in albumin uptake [59, 60]. The uptake of albumin in Megalin-, Cubilin- and double-knock out mice was completely inhibited that indicates these receptors are needed for the uptake of albumin [59, 60].

In a study by Weyer et al. using Megalin/Cubilin deficient mice, ^125^I-labelled murine albumin was used to investigate the uptake in the kidney and urinary excretion of intact albumin as well as its fragments by using size-exclusion chromatography [61]. For control mice all albumin was eluted as fragments, whereas the Megalin/Cubilin-deficient mice showed a decreased albumin uptake in the kidneys, as well as decreased degradation, together with an increased excretion of intact labelled albumin. An albumin conjugate, only fluorescent when intracellularly degraded, was used to visualize the degradation in proximal tubular cells after intravenous injection. Proximal tubular cells in control mice were positive, while there was an absence of fluorescence in Megalin/Cubilin deficient mice that indicated an Megalin/Cubilin-mediated endocytosis mechanism also plays a role in the intracellular degradation of albumin in the proximal tubular cells [61]. Furthermore, a study by Zhai et al. using a double labelling strategy of fluorescent albumin and antibodies against either of the two receptors Megalin or Cubilin, showed a correlation between Megalin and Cubilin expression and the uptake of albumin that supports a role in reabsorption of albumin [62].

### Neonatal Fc receptor (FcRn)

A major role of the neonatal Fc receptor (FcRn) is in placenta and proximal small intestine transport of IgG from mother to fetus [63]. FcRn is a glycoprotein comprising of a MHC-class I-like heavy chain, consisting of three extracellular domains (α1, α2, and α3), which is non-covalently associated with a β_2_-microglobulin (β2m) light chain. The light chain is necessary for the function of FcRn [64]. The heavy chain is connected to a transmembrane element that continues into the cytoplasm.

It has been revealed that a lower amount of Immunoglobulin G (IgG) antibodies were present in the blood of β_2_m deficient mice and that immunization of the mice showed decreased immune responses probably due to degradation of IgG caused by a lack of diversion from lysosomal degradation facilitated by FcRn [65–67]. This indicates, therefore, that FcRn plays a role in adults as well as in the neonatal state. FcRn is distributed in many tissues including vascular endothelium as well as the gut, lungs and kidney [63]. The first evidence for albumin/FcRn binding was co-elution of bovine albumin and soluble human FcRn on a human IgG-coupled column [28], also suggesting that both IgG and albumin could simultaneously bind FcRn. Work by the same group revealed that the serum concentration of albumin in FcRn deficient mice was reduced compared to wild-type mice and that FcRn-deficient mice had shortened half-life of albumin [28].

Domain III was first suggested as the primary binding site for FcRn [68, 69]. However, a study of FcRn binding to recombinant domain III alone showed a ten-fold weaker FcRn binding compared to non-recombinant albumin [68, 70]. In the same study a docking model of human FcRn in complex with human albumin revealed FcRn interactions with two loops in the N-terminal of domain I, in addition to the interactions in domain III [68]. Site-directed mutagenesis of specific residues residing in the loops in domain I resulted in an altered affinity to FcRn [71]. Co-crystallization studies of human FcRn in complex with human albumin supports involvement of both domain III and domain I in FcRn binding [72, 73]. In vitro studies have shown that albumin binding is dependent on the presence of a conserved histidine residue in FcRn (His166) [74, 75]. X-ray crystallography data revealed a loop surrounding the His166 at acidic pH. Hence, the theory of a pH-sensitive loop stabilized by the protonated His166 was proposed [76, 77]. Furthermore, the loops were shown to contain four conserved tryptophan residues that resulted in reduced or loss of binding to albumin when mutated [72, 78]. This indicates that the binding of albumin is not only pH dependent but also hydrophobic and that both domain I and III are involved in FcRn interaction.

A cellular FcRn-mediated recycling pathway was first proposed for IgG by Brambell in 1965 [79]. Later the hypothesis that albumin recycling was carried out by the same mechanism was proposed [80]. It is widely accepted that FcRn is responsible for IgG half-life extension by a mechanism of increased binding at low pH (<6.5) within the endosomes and recycling and release into the extracellular space at physiological pH. The first indications for FcRn involvement in albumin recycling were revealed in 2003 by Chaudhury et al. [28]. The authors confirmed the hypothesis of a single receptor responsible for the half-life regulation of albumin in the same manner as for IgG [81] by showing FcRn-albumin binding and a shortened life-span of albumin in FcRn-deficient mice.

### Albumin-based drug delivery strategies

**Fig. 2:**
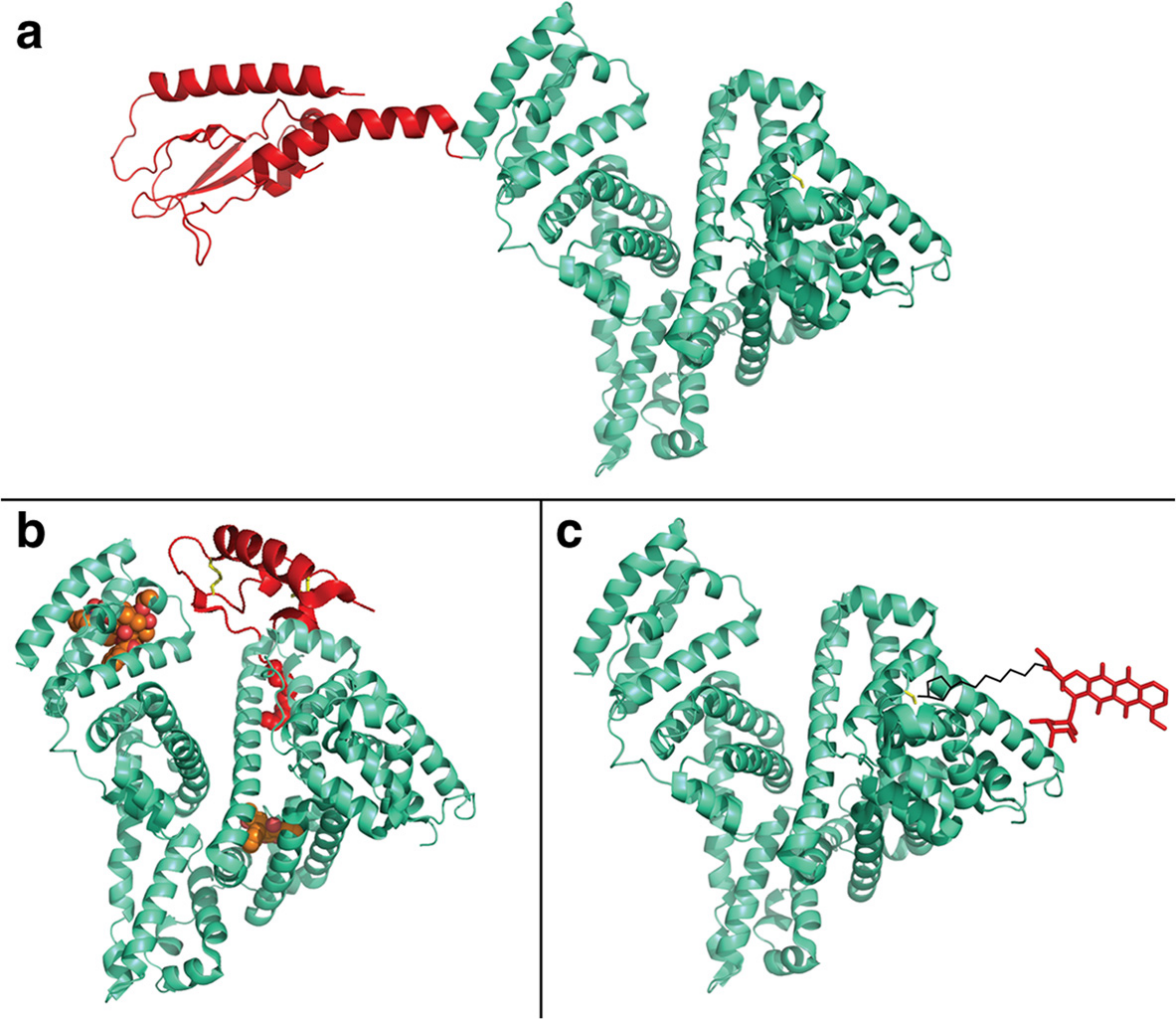
Albumin-based drug delivery strategies. **a** Albumin fusion-based drugs, in light green (HSA) and in red (fusion peptide) (modified PDB 1e7e + 3IOL). **b** Albumin associating drugs; upper left binding of paclitaxel (from PDB1JFF), upper right binding of insulin detemir (Levemir®) (insulin from PDB1ZNI, Myristic acid from PDB1H9Z), lower panel binding of the weakly associated warfarin (PDB1H9Z). **c** Covalent conjugation of a drug to albumin via the available Cys34 (modified PDB 1e7e + 1I1E)

The natural transport function, multiple ligand binding sites, and cellular interactions provides rational for the exploitation of albumin for drug delivery. The ability to covalent and non-covalently attach drugs or expression of albumin-drug fusions provides a range of design options (Fig. 2) that has been taken into clinical trials or on the market (Table 1).

**Table 1:** A selection of albumin-based systems in clinical trials and marketed products

Attachment	Name	Disease	Drug type	Clinical status	Company	Ref
Non-covalent/reversible association	Levemir®	Diabetes type 1 and 2	Insulin detemir	Marketed	Novo Nordisk	[123, 124]
	Victoza®	Diabetes type 2	GLP-1	Marketed	Novo Nordisk	[123]
	Ozoralizumab	Rheumatoid arthritis	Antibody derivative	Phase II completed	Ablynx	[90]
Covalent	MTX-HSA	Cancer and autoimmune diseases	Methotrexate	Phase II	Access Pharmaceuticals Inc.	[26, 119, 125, 126]
	Aldoxorubicin	Cancer	Doxorubicin	Phase I completed	CytRx, Inc.	[110, 127]
	CJC-1134	Diabetes type 2	Exendin-4	Phase II	ConjuChem	[11, 128–131]
Genetic fusion	Eperzan/Tanzeum	Diabetes type 2	GLP-1	Marketed	Glaxo Smith Kline	[132–134]
	N/A	Hemophilia	FVIIa	Phase I completed	CSL Behring GmbH	[135–138]
	N/A	Hemophilia B	rIX-FP	Phase III completed	CSL Behring GmbH	[139]
	Albuferon®/Zalbin/Jouleferon	Hepatitis C	INFalpha-2b	Phase III completed, Development ceased	Human Genome Sciences in collaboration with Novartis	[11, 140]
Micro-/Nanoparticle	Abraxane®	Cancer	Paclitaxel	Marketed	Celgene	[141]
	ABI-008	Cancer	Docetaxel	Phase I/II	Celgene	[95]
	ABI-009	Cancer	Rapamycin	Phase I/II	Celgene	[96]
	ABI-010	Cancer	HSP90 Inhibitor	Withdrawn before enrollment	Celgene	[97]
	^99m^Tc-Albures	Diagnostic purpose	Technetium-99	Marketed	GE Healthcare	
	^99m^Tc-Nanocoll	Diagnostic purpose	Technetium-99	Marketed	GE Healthcare	

### Albumin-associated drugs

Albumin binds to endogenous ligands such as fatty acids; however, it also interacts with exogenous ligands such as warfarin, penicillin and diazepam. As the binding of these drugs to albumin is reversible the albumin-drug complex serves as a drug reservoir that can enhance the drug biodistribution and bioavailability. Incorporation of components that mimic endogenous albumin-binding ligands, such as fatty acids, has been used to potentiate albumin association and increase drug efficacy. Examples include Levemir® (Insulin detemir) and Victoza® (Liraglutide) manufactured by Novo Nordisk for the treatment of diabetes. Levemir® is a myristic acid modified insulin analog. While for Victoza® a palmitic acid is attached to a glucagon-like peptide-1 agonist. On injection the fatty acid moiety binds to albumin and dissociates over time and, therefore, enhances the bioavailability and distribution. Levemir® has been shown to improve glycaemic control and resulted in limited serious adverse drug reactions that was evaluated in a large multi-national follow up data study after 14 weeks in which the safety and efficacy was assessed of 20,531 patients with type 1 or 2 diabetes [82]. Victoza® went through 8 phase III trials to evaluate the efficacy and safety of Victoza® as a monotherapy or as a combination therapy. Victoza® resulted in improvements in both hemoglobin A_1c_ and fasting plasma glucose (FPG) [83–89]. Benefits of those insulin analogues by albumin-binding are an extended time of action profile compared to conventional basal insulin such as neutral protamine Hagedorn (NPH) that peak before 8 h of injection [82].

Another category that utilises specific-binding to albumin is nanobodies. Ablynx has developed ATN-103, now known as Ozoralizumab, which is a trivalent antibody having two peptides, one to interact with TNF-α, and the other, albumin. In collaboration with Pfizer, Ozoralizumab has completed Phase II studies in patients with rheumatoid arthritis [90]. Five different dosing groups were compared to placebo treatment and the highest dose of Ozoralizumab (80 mg every 4 weeks) improved the ACR20 response compared to placebo in week 16 [91].

An alternative strategy to specific ligand binding is non-specific association of albumin. Albumin has hydrophobic binding domains in which drugs such as warfarin and diazepam can bind. Abraxane® is an established albumin-based nanoparticle system produced by Celgene and is used in the treatment of cancer. It is proposed to be an albumin-bound nanoparticle of about 130 nm in which the outer layer consists of albumin while the inner core contains the water insoluble cytotoxic agent paclitaxel [92]. It has been shown to be less toxic to its free drug counterpart paclitaxel and also exhibits higher anti-tumour activity compared to free paclitaxel [92]. SPARC has been hypothesized to support tumour uptake of Abraxane®. A preliminary study showing that SPARC-positive cancer patients had a higher response to an Abraxane®, supports the hypothesis that SPARC mediated accumulation of albumin in tumours increases the effectiveness of albumin-bound paclitaxel [54]. In contrast, a study from 2014 on genetically modified SPARC-deficient mice did not show any difference in uptake of Abraxane® into tumours [93]. The uptake mechanism of Abraxane® in cells remains to be elucidated, yet, Desai et al. have proposed that Gp60 and SPARC work in combination [54] suggesting Abraxane® is transported across the endothelial barrier by binding to Gp60 and subsequent caveolae-mediated transcytosis into the tumour interstitium where SPARC enhances the uptake of Abraxane into tumour cells [54]. Celgene has a portfolio of albumin-based nanoparticles for cancer treatment, which have been presented in a report by Desai [94]. In this report, preclinical studies of ABI-008 and ABI-009 are described. ABI-008 contains the active drug docetaxel. It has completed phase I/II [95] and showed anti-tumour effects in preclinical studies using xenograft studies of prostate and colon tumours as reviewed by Desai [94]. Likewise, ABI-009 in which the active drug is rapamycin proved to be effective against colon and breast tumours in xenograft studies and exhibited low toxicology and good efficacy [94]. To our knowledge it has reached a combined phase I and phase II study in the treatment of non-muscle invasive bladder cancer [96]. ABI-010 contains a Hsp90 inhibitor 17-allylamino-17-demethoxygeldanamycin (17-AAG). Hsp90 is a chaperone that helps to fold signaling proteins involved in cancer; hence, it is an interesting candidate for cancer treatment. A phase I trial was planned for ABI-010 in a combination treatment with Abraxane® for different hematological malignancies though it has been withdrawn prior to enrollment [97].

In addition to albumin-based nanoparticle therapeutics, diagnostic nanoparticles have been developed. ^99m^Tc-Albures and ^99m^Tc-Nanocoll are both albumin aggregated particles containing the metastable nuclear isotope of technetium-99 that have been used for various diagnostics purposes in cancer and infectious diseases [98, 99]. In a study of 59 patients with peripheral joint pain, ^99m^Tc-nanocolloid scintigraphy showed that the scan was able to detect 82 % of the clinically assessed joint disease in a group with arthralgia [100]. In a study of rheumatoid arthritis comparing clinical assessment with ^99m^Tc-nanocolloid scans, 79 % of clinically positive joints were detected by the scan [101].

### Albumin-fusions

An elegant approach to combine protein-based drugs with albumin, is genetically fusion to the N- or C-terminal or both ends of the albumin. The protein gene is connected to that of albumin and expressed in a suitable expression host, typically yeast, resulting in a single fused protein. It is, however, necessary that the linker and fused moiety do not interfere with the folding of albumin so it retains its functionality and long half-life.

The product albiglutide (Eperzan®/Tanzeum®) manufactured by GlaxoSmithKline for the treatment of type II diabetes, is a GLP-1 receptor agonist developed by fusion of two human GLP-1 repeats to recombinant human albumin [102, 103]. In eight phase III studies also known as the Harmony program, the efficacy and safety profile of albiglutide has been studied. A detailed review by Woodward et al. shows that weekly dosing of albiglutide showed lowered glycated hemoglobin, reductions in fasting plasma glucose and weight loss in patients with type II diabetes [104].

Albuferon®, also known as albinterferon, is an interferon α-2b fused to albumin that went into phase III studies for treatment of Hepatitis infections. In the phase IIb study of a combination therapy of ribavirin and albinterferon to treat hepatitis C virus, patients given albinterferon of 900 μg and 1200 μg every 2 weeks showed the same sustained virologic response as the standard treatment of PEGylated interferon α-2a (Pegasys®) 180 μg every week [105]. In the phase III studies albinterferon was equal to standard treatment of PEGylated interferon α-2a though treatment discontinuation due to adverse effects which were 4.1 %, 10.4 % and 10.0 % for PEGylated interferon α-2a, albinterferon 1200 μg and albinterferon 900 μg respectively [106, 107]. As of October 2010 FDA issued a complete response letter and Novartis and Human Genome Sciences, Inc. decided to stop further development of the drug [108].

### Covalent attached drugs

A standard approach is chemical conjugation of the drug to either lysines, tyrosines, or the free SH-group on the cys34. The free thiol group on cys34 has been widely used, for instance by reacting with a maleimide linker from prodrugs, which have been intravenously injected [109, 110]. Covalent attachment of drugs, however, requires a release mechanism from albumin. In the group of Kratz, this was solved by introducing an acid sensitive hydrazone linker that is thought to be cleaved upon delivery at tumour sites due to an acidic extracellular environment or inside endosomes or lysosomes after cellular uptake [11, 109]. The group of Kratz modified doxorubicin with maleimides and demonstrated in situ conjugation with cys34 of endogenous albumin after intravenous injection. This is based on 70 % of the endogenous pool of albumin contributing to free thiols. In vivo studies performed by the same group revealed that doxorubicin maleimide derivatives were superior to free doxorubicin with regards to anti-tumour efficacy and toxicity in three different animal models (RENCA, MDA-MB 435 and MCF-7) [111, 112]. This work by the group of Kratz was taken further and Aldoxorubicin (also known as INNO-206 or DOXO-EMCH) produced by CytRx is a doxorubicin conjugate containing an acid-sensitive linker. Upon administration the linker is thought to bind to circulating albumin and is, therefore, transported to the tumour site where the acidic environment will cleave the linker and release doxorubicin to exert its action. Aldoxorubicin was shown to be superior to doxorubicin in a Phase IIb study involving 126 patients for treatment of soft tissue sarcoma [113]. CytRx has initiated a phase III global trial of their anti-cancer drug Aldoxorubicin for soft tissue sarcoma, and phase II studies and below are ongoing for treatment of small cell lung cancer, HIV-related Kaposi’s sarcoma and late-stage glioblastoma [114]. CytRx are also studying Aldoxorubicin combination treatments, for instance Ifosfamide for patients with soft tissue sarcoma and Gemcitabine to treat metastatic solid tumours [114].

Lau et al. used maleimide conjugation to link small interfering RNA (siRNA) to endogenous albumin. Using SMCC, a thiol-reactive group was incorporated terminally in the siRNA able to react to the free cys34 on circulating albumin [115]. Ex vivo results indicated a fast reaction of maleimide-activated siRNA with cys34 on albumin, and after 1 h maximal conjugation was reached. Furthermore, in vivo work showed that siRNA-albumin was still detectable after 4 h, whilst non-activated siRNA was not after 30 min [115]. In vivo silencing of mice treated with activated siRNA (1 mg/kg) resulted in significantly reduced levels of the myocardium target gene IGF-IR mRNA compared to vehicle treated or nonactivated siRNA [115]. Hence, siRNA-albumin conjugates may be useful for gene silencing in tissues.

Ehrlich et al. have conjugated an Y2R-peptide to albumin to enhance its circulation time [116]. The Y2R-peptide is a potential obesity drug as it acts on the Y2 receptor located in the hypothalamus and peripheral nervous system and is, therefore, thought to reduce appetite. The Y2R-peptide was modified using different linkers (succinimidyl 4-[N-maleimidomethyl]cyclohexane-1-carboxylate (SMCC), 6-maleimidohexanoic acid N-hydroxysuccinimide ester (MHS), and N-[γ-maleimidobutyryloxy]-sulfosuccinimide ester (GMBS) before attachment to albumin. One of the most active albumin conjugates in vitro (HSA-MH-Y2R) showed a significant reduction in food uptake after 24 h of 37 % [116].

Methotrexate human serum albumin (MTX-HSA) is a covalent attached methotrexate to lysine residues in albumin. In a study by Stehle et al. it was found that the drug loading ratio to albumin affected the tumour targeting properties in a rat tumour model [117]. Though, it was thought that more MTX attached to HSA would increase the therapeutic effect, it was found that a low molecular ratio of 1:1 resulted in the highest tumour targeting properties such as high tumour uptake, long half-life and low liver uptake rates [117]. Phase I studies of MTX-HSA in cancer patients applied at a ratio of 1:1.3 did not result in any severe side-effects and was in general well tolerated by the patients, therefore, showing a good toxicology profile [118]. MTX-HSA was used in combination with cisplatin in treatment of patients with bladder cancer in a phase II study. One patient showed a partial response and another showed complete response out of seven patients resulting in 27 % response rate [119]. To our knowledge, MTX-HSA has not been taken further for clinical studies.

## Conclusion and future perspectives

Exploitation of the natural properties of ligand binding and transport have been utilised for albumin-based drug delivery, with a focus on drug half-life extension. A drug construct design incorporating binding ligands is a simple, but elegant, approach used for commercial reversible binding drugs Levemir® and Victoza®. A more elaborate non-reversible strategy is development of albumin covalent conjugated drugs. The availability of a free thiol at cys34 in domain I allows site-specific conjugation distant from the main FcRn binding site in domain III and Hyphenate if the word is on two lines binding pockets, a chemoselectivity not possible when conjugation is performed to the multiple lysines distributed throughout albumin. Thiol-maleimide conjugation is the dominant method employed to attach drugs; however, the susceptibility of the maleimide bond to serum breakdown in the bloodstream due to thiol exchange reactions may require alternative chemistries [120]. Pre-hydrolysis of the maleimide-conjugate prior to thiol exposure to create a stable open-ring structure is a promising approach [121]. The application of albumin fusions containing a therapeutic protein is a strategy that circumvents the requirement for covalent conjugation. Eperzan®/Tanzeum® is now on the market, with the number of albumin fusion products expected to rise. The application of engineered recombinant albumins with different affinity to FcRn shown in non-human primates to tune the drug pharmacokinetic profile is an exciting next-generation approach [122]. Interaction with a range of cellular receptors such as Gp18, Gp30 and Gp60 may potentiate cellular entry for intracellular drug delivery applications. A greater understanding of the intracellular pathway of albumin, however, is needed in order to optimise albumin-based intracellular drug delivery approaches.

Albumins inherent transport properties and cellular receptor engagement promotes albumin as a natural molecular medicine, greater control of these properties is key to further harness nature to cure disease.
